# LDmat: efficiently queryable compression of linkage disequilibrium matrices

**DOI:** 10.1093/bioinformatics/btad092

**Published:** 2023-02-16

**Authors:** Rockwell J Weiner, Chirag Lakhani, David A Knowles, Gamze Gürsoy

**Affiliations:** Department of Biomedical Informatics, Columbia University, New York, NY 10032, USA; New York Genome Center, New York, NY 10013, USA; Department of Computer Science, Columbia University, New York, NY 10027, USA; New York Genome Center, New York, NY 10013, USA; New York Genome Center, New York, NY 10013, USA; Department of Computer Science, Columbia University, New York, NY 10027, USA; Department of Systems Biology, Columbia University, New York, NY 10032, USA; Department of Biomedical Informatics, Columbia University, New York, NY 10032, USA; New York Genome Center, New York, NY 10013, USA; Department of Computer Science, Columbia University, New York, NY 10027, USA

## Abstract

**Motivation:**

Linkage disequilibrium (LD) matrices derived from large populations are widely used in population genetics in fine-mapping, LD score regression, and linear mixed models for Genome-wide Association Studies (GWAS). However, these matrices can reach large sizes when they are derived from millions of individuals; hence, moving, sharing and extracting granular information from this large amount of data can be cumbersome.

**Results:**

We sought to address the need for compressing and easily querying large LD matrices by developing LDmat. LDmat is a standalone tool to compress large LD matrices in an HDF5 file format and query these compressed matrices. It can extract submatrices corresponding to a sub-region of the genome, a list of select loci, and loci within a minor allele frequency range. LDmat can also rebuild the original file formats from the compressed files.

**Availability and implementation:**

LDmat is implemented in python, and can be installed on Unix systems with the command ‘pip install ldmat’. It can also be accessed through https://github.com/G2Lab/ldmat and https://pypi.org/project/ldmat/.

**Supplementary information:**

Supplementary data are available at *Bioinformatics* online.

## 1 Introduction

Linkage disequilibrium (LD) is a measure of how often alleles at different loci appear together in a population (Collins, 2007). There are several alternative methods of computing LD between two loci, all of which provide values between −1 and 1 (Kijas *et al.*, 2014; Mueller, 2004; Myers *et al.*, 2020). High LD values between two alleles correspond to them occurring together frequently in the population and consecutive regions of the genome with high LD values are dubbed haplotype blocks or haploblocks (Gabriel *et al.*, 2002). These haploblocks are known to be associated with hotspots of recombination (Slatkin, 2008). Along these lines, LD can provide powerful insights into population genetics, as these values are associated with natural selection, genetic drift, and other genome altering events (Cutter, 2019; Ennis, 2007; Hudson, 2004). LD scores are used in fine-mapping, LD score regression, and linear mixed models for Genome-wide Association Studies (GWAS). Since LD values can be calculated between every pair of variants in a chromosome, matrices are the most natural representation. However, even for a small chromosome, the total number of distinct data points is on the order of 10^15^. Typically, these values are only significantly non-zero for loci which are somewhat close to one another (*i.e.*, in the same haploblock), so only nearby values may be calculated in practice. However, this only reduces the total number of data points by a few orders of magnitude, depending on the chosen genomic distance. For example, LD matrices calculated using the genotypes in the UK Biobank (UKBB) are publicly available in compressed numpy array format (Harris *et al.*, 2020) and the total size of the data ranges from approximately 45 GB (chromosome 21) to 250 GB (chromosome 2), even with values given only for pairs of SNPs or indels that are within 3 MB of each other (Weissbrod *et al.*, 2020). Moving, sharing and extracting granular information from this large amount of data can be cumbersome. Compounding the problem, there is no standard file format for storing these LD matrices. This means that LD matrices for different cohorts often use different *ad hoc* formats [e.g. LDStore2 (Benner *et al.*, 2017) or Hail] and custom downstream analysis tools are required. Both the large file size and lack of standardization make it difficult to extract useful information from these files. Extracting a small sub-matrix from these large matrices requires access to large resources capable of storing all of the data in memory, along with a bespoke script to find and query the appropriate file(s) containing the relevant information. In particular, the memory to read these large files and file IO time become an important problem in downstream analysis. This prevents scientists with scarce resources from accessing and working on LD matrices from large population genetics studies, hence hampering advances in biomedical research. To address these issues, we developed a user-friendly tool called LDmat that can effectively compress LD matrices with an up to 90% compression rate. We also provide functionality that can query compressed LD matrices by desired loci and minor allele frequency (MAF) threshold and visualize the resulting sub-matrices. This tool is similar to TABIX (Li, 2011), but works with matrix format.

## 2 Ldmat functionalities

The tool includes two main modules.

### 2.1 Compress

**Fig. 1.**
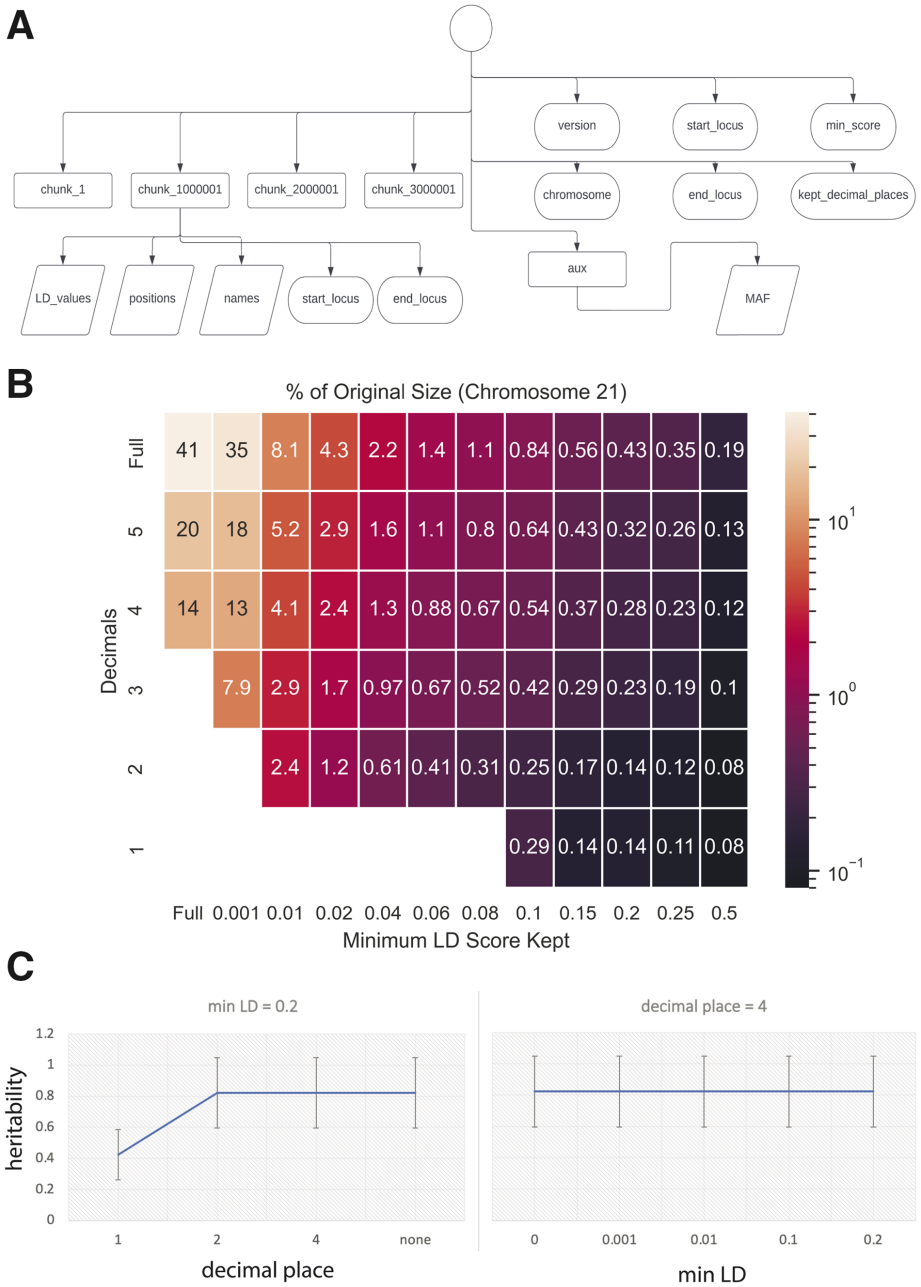
(**A**) Internal structure of the HDF5 file. Rectangles represent groups, which are the HDF5 equivalent of dictionaries. The datasets and metadata attributes are shown in detail for ‘chunk_1000001’ only, although all chunks have the same set of entries. These chunks correspond to the trapezoids in Supplementary Figure S2. (**B**) The size of the compressed files expressed in terms of the percentage of the size of the original files at different parameters. ‘Lossless’ is shown as ‘full’. (**C**) Heritability of the height trait in UKBB is calculated from resulting LD matrices after truncation with keeping different decimal places and minimum LD thresholds

We used Hierarchical Data Format version 5 (HDF5) for our compression mechanism. LDmat can compress a large LD matrix and associated MAF values (optional) down to a single HDF5 file (see Supplementary Information). Within this file, there exist many ‘groups’, each one covering a non-overlapping section of chromosome positions (Fig. 1A). These groups contain pointers to the data arrays indexed in HDF5. An appropriate group size is chosen automatically based on the overlap in the input files (although a different size can be manually specified). For the UKBB .npz files, the automatically chosen size is 1 MB.

### 2.2 Query

When making a query, the tool must first find the groups within the HDF5 file that contain the desired data by checking for overlap with the start and end locus of each group (see Supplementary Information). In order to accommodate large queries without running out of memory, the tool can write the results to disk as they are calculated. This feature turns on automatically when the queried sub-matrix passes a size threshold.

## 3 Results

### 3.1 Compression and querying

We ran a series of tests on Chromosome 21 (and Chromosome 1, see Supplementary Information), compressing the full set of UKBB LD matrices into a single file. The total size of the raw data is 45,185 MB. In Figure 1B (see Supplementary Fig. S4 for Chromosome 1), we have the results of compressing these files, while varying the minimum LD value and decimal place parameters.

Notably, the data can be compressed down to less than 1% of its original size, when four decimal places and absolute LD value threshold of 0.06 are kept (Supplementary Fig. S5). Note that lossless compression with HDF5, that is, when all decimal places and LD values are retained, results in 59% reduction in file sizes (Fig. 1B). These compressed files also include the MAF values, which are not present in the original LD matrices. Furthermore, if we apply the same decimal places and minimum LD value threshold to the original .npz files, they are still over 3-fold larger than the corresponding HDF5 files. Moreover, they do not contain any metadata or auxiliary data such as the MAF values. We calculated the compression ratio as a function of minimum LD score threshold and found that compression rate plateaus for threshold values larger than 0.06 (Supplementary Fig. S5a), which can provide guidance on how to select the threshold. We also tested the computational time of running a set of randomized queries on our compressed LD matrices. We showed that LDmat query functionality can return a sub-matrix of 1 MB locus under 2 seconds when tested on both Chromosomes 1 and 21 (Supplementary Table S1). The query runtime for consecutive loci is dependent on the group sizes in HDF5. If the queried locus is larger than the group size, then LDmat has to search more than one hash table, increasing the time to query. To demonstrate how this works, we created a compressed matrix from Chromosome 21 with a group size of 0.5 MB. We then showed that querying a 1 MB locus in this matrix takes two times longer compared with a matrix with group size of 1 MB (2.4 seconds versus 1.25 seconds, Supplementary Table S1). We also showed that it takes around 2 and 6 min to return LD scores between a list of 10^6^ non-consecutive loci on Chromosomes 21 and 1, respectively. See Supplementary Information for examples and Supplementary Figure S6 for usage.

### 3.2 Utility

In order to assess the accuracy of the compressed LD matrices, we first looked at the distribution of LD values after removing scores that are below the LD value threshold. As expected, the overall distribution and statistics of the LD values remain the same, while the number of LD values that are zero (which is the mean in all cases) changed (Supplementary Fig. S7). We then calculated the heritability of the height trait using UKBB data for different minimum LD value and decimal place thresholds using LD score regression (Bulik-Sullivan *et al.*, 2015). We found that both heritability estimate and the standard error on heritability estimate (Fig. 1C) are significantly affected if we keep less than three decimal places, while they remain the same at every minimum LD value threshold up to 0.2. We also observed the same when we compared the LD score regression coefficients of annotations per thresholds (Supplementary Fig. S8). This is because while the minimum LD threshold affects only a subset of LD values, the decimal place threshold affects all of the LD values.

## 4 Conclusions

In conclusion, we recommend users to set the decimal place threshold to 4 and minimum LD value threshold to 0.1 for most accurate results. This will still result in shrinking the LD matrices to ∼1% of their original size (Fig. 1B). Since the compression rate is large even when we use very small minimum LD value thresholds and a large number of decimal places, we recommend users to optimize their choices based on the utility, that is, the minimal information loss.

## Supplementary Material

btad092_Supplementary_Data

## Data Availability

The code and the test data can be accessed through https://github.com/G2Lab/ldmat.
