# Supplementary material for: LDmat: efficiently queryable compression of linkage disequilibrium matrices
Source: Bioinformatics. 2023 Feb 16;39(2):btad092. doi: 10.1093/bioinformatics/btad092 (PMC9969815; doi:10.1093/bioinformatics/btad092)
Supplement: btad092_Supplementary_Data [file btad092_supplementary_data.pdf]

Supplementary Material

Input file format

Despite the tool being developed to serve this specific data format, it has quite a bit of additional flexibility. First, the name of the files need not match '$chrA\_B\_C.npz$', as long as the start and end locus are contained somewhere in the name. This is the use case for the '–locus-regex' option. Additionally, the submatrices need not be overlapping. The tool also natively supports other file formats besides 'npz', such as CSV and TSV. In these cases, there is no need for a metadata file, as the row and column names can be included in the data files themselves. Perhaps most importantly, ldmat also makes it simple to write custom data handlers, so that any file format can be used.

Compression Functionalities

In practice, LDmat can take any file type in matrix form as input . However, here we describe how it works with .npz files since LD matrices are oftentimes stored in numpy array format. For example, the UKBB LD matrix is divided into multiple submatrices (Weissbrod *et al.*, 2020). Each submatrix contains LD values for the SNPs in a 3Mb window (only the lower triangle due to symmetry) and these windows are slid along each chromosome in 1Mb intervals (see depiction in Fig. S1). Each of these .npz files only contain LD values, while the corresponding genomic coordinates are stored in a separate compressed file. There are four main steps by which LDmat reduces the original file size. (1) The highest compression level in HDF5 is applied (same level as gzip). However, this is not a significant improvement if starting with the .npz files, as they are already saved with this compression level. (2) All redundant data, i.e., overlapping submatrices, is removed. We changed the format from upper triangles to smaller trapezoids (Fig. S2) (3) We remove the LD values whose absolute values are smaller than a user specified threshold value as they may not be informative about the association between alleles or may be noise, and (4) We remove decimal places that are larger than a user specified threshold value. LDmat outputs a single HDF5 file, which is a flexible format allowing for a hierarchical "file" structure within the file.

All necessary metadata such as start and end coordinates are stored along with each group, as HDF5 "attributes" (Fig. 1a). The auxiliary data such as MAFs are also stored in their own group.

convert: convert takes a single file containing an LD matrix (any file format [txt or npz] and compresses it to the HDF5 format. The user can specify the minimum LD value and decimal points to keep.

convert-chromosome: convert-chromosome takes a path to a set of files containing sub matrices of the same chromosome (such as the format of UKBB LD matrices) and compresses them to the HDF5 format. The user can specify the minimum LD value and decimal points to keep.

convert-maf : convert-maf takes a file containing MAF values as input, and adds them to an existing HDF5 file as auxiliary data. This is required for some of the query capabilities such as submatrix-by-maf command (see below).

Query Functionalities

If the desired data spans multiple groups within the HDF5 file, the resulting matrix is constructed piece by piece, moving from upper left to bottom right. Since the matrices are symmetrical, no data below the diagonal is stored, thus requiring the tool to transpose data from the upper triangle where appropriate.

submatrix: submatrix takes an HDF5 file as input, along with the desired range of locus start and end positions to select as a submatrix. For small matrices, the results are printed. For larger matrices, it is recommended to specify an output file. For extremely large matrices, the output will be streamed to a file in several chunks to avoid memory issues. Finally, the resulting matrix can be plotted as a heatmap (Figure S3).

submatrix-by-list submatrix-by-list works similarly to submatrix, but takes as input a list of desired locus positions, specified in a text file.

submatrix-by-maf: convert-chromosome also works similarly to submatrix, but takes a lower and upper bound of desired MAF values as arguments. The command finds all loci in the chromosome whose MAF values are within the range, and then returns the submatrix consisting of all such loci (as both the rows and columns).

3.0.1 Query Time

: See Table S1. As expected, range queries are much more efficient, and generally complete in less than a second for ranges under 10^5 bp. The relatively slow performance of list queries comes from the fact that nearly all data groups will be involved in any given query (as opposed to just a few for a range query), and that random access to an array is an inherently slower operation than selecting a contiguous portion.

Usage Examples

Reconstructing Original Files

An informative but not very useful sequence of actions lets us compress the original set of files down to a single HDF5 file, and then reconstruct any of the original files from this new one. Suppose we wish to work with chromosome 1, whose files are stored as

`foo/chr1_1_3000001.npz, foo/chr1_3000001_6000001.npz, etc.`

First, we create the lossless single file with:

```
ldmat convert\-chromosome foo/chr1_*.npz chr1.h5 \-c 1
```

This creates a single file called "chr1.h5". Now, given only this new file, we can recreate the original $foo/chr1\_1\_3000001.npz$ with:

```
ldmat submatrix -rs 1 -re 3000001 -o bar/chr1_1_3000001.npz
```

Except for some very small (less than 10-6) rounding errors, $foo/chr1\_1\_3000001.npz$ and $bar/chr1\_1\_3000001.npz$ will be identical.

LD matrices as input to SuSiE

One motivating use case for the development of this tool is the need for LD scores to run SuSiE (Wang *et al.*, 2020). Previously, to have LD matrices for a select set of Quantitative Trait Loci (QTLs) from the many LD files, special handling was needed to find the appropriate file, load the data, and select only the desired loci. And most problematically, this could only be performed on a large cluster with space to store the huge amount of data comprising these files.

Now, one can simply save the list of QTLs to a file, and select them with:

```
ldmat submatrix-by-list chr1.h5 -r QTL_list.csv -o LD_out.csv
```

In practice, one would often want to avoid writing and reading files, and instead simply choose to import the python package and call the methods directly, passing a list of loci and receiving a Pandas Dataframe as the result.

Supplementary Tables

**Size of the query range (in bp)**	1	10	10^2	10^3	10^4	10^5	10^6	**Chr**	**Group size**
Range queries with a random start position	0.01	0.01	0.01	0.01	0.01	0.01	1.25	Chr 21	1 MB
Random list of loci	0.01	0.20	0.82	1.08	3.07	21.60	119.94	Chr 21	1 MB
Range queries with a random start position	0.01	0.01	0.01	0.01	0.01	0.04	2.41	Chr 21	0.5 MB
Random list of loci	0.01	0.21	1.31	2.18	3.80	19.57	134.93	Chr 21	0.5 MB
Range queries with a random start position	0.02	0.01	0.02	0.02	0.02	0.04	1.34	Chr 1	1 MB
Random list of loci	0.01	0.21	1.86	5.82	8.35	41.15	368.26	Chr 1	1 MB

Table 1. Time (in milliseconds) it takes to run queries of different sizes. Range queries with a random start position refers to a set of consecutive loci with a random start location. Random list of loci refer to a set of random number of loci that are not necessarily consecutive. These queries were run on a MacBook Air with an Apple M1 Chip and 8GB of memory.

	d = none	d = 4	d = 2	d = 1
m = 0	**0.8226 (0.2269)**			
m = 0.001		**0.8226 (0.2269)**		
m = 0.01		**0.8226 (0.2269)**	0.8204 (0.2259)	
m = 0.1		**0.8226 (0.2269)**	0.8204 (0.2259)	0.4237 (0.1611)
m = 0.2		**0.8226 (0.2269)**	0.8204 (0.2259)	0.4237 (0.1611)

Table 2. The heritability and the standard error in heritability for different minimum LD value (m) and decimal place (d) cut-offs

Supplementary Figures

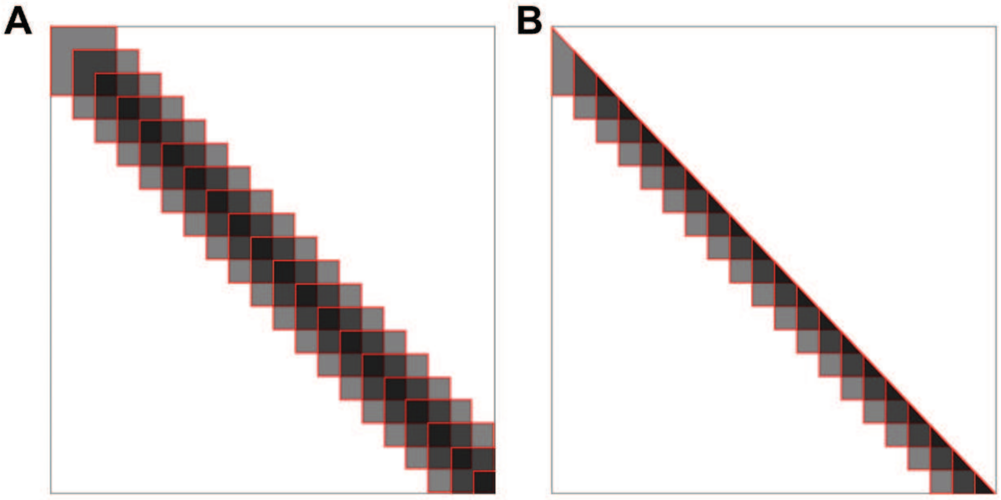

Fig. 1. (A) Diagram of the full matrix for a chromosome in the .npz format, which consists mainly of empty space since only LD values near the diagonal are calculated. Each square submatrix shows all the data that is actually covered by the npz files. (B) Same as (A), but shows only the lower triangle of each submatrix, since those are the values actually stored in the npz file.

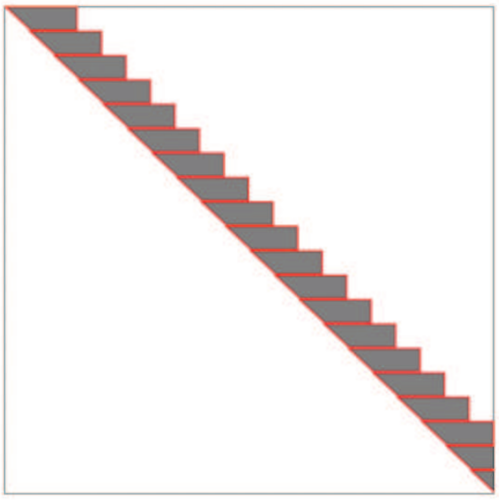

Fig. 2. Depiction of the groups comprising the compressed HDF5 file created by LDmat. These groups are nonoverlapping, yet contain all of the data originally present in the npz files. Additionally, they are transposed to contain only portions of the upper triangle of each submatrix. The transposition is not strictly necessary, but makes the logic of querying and indexing simpler to follow.

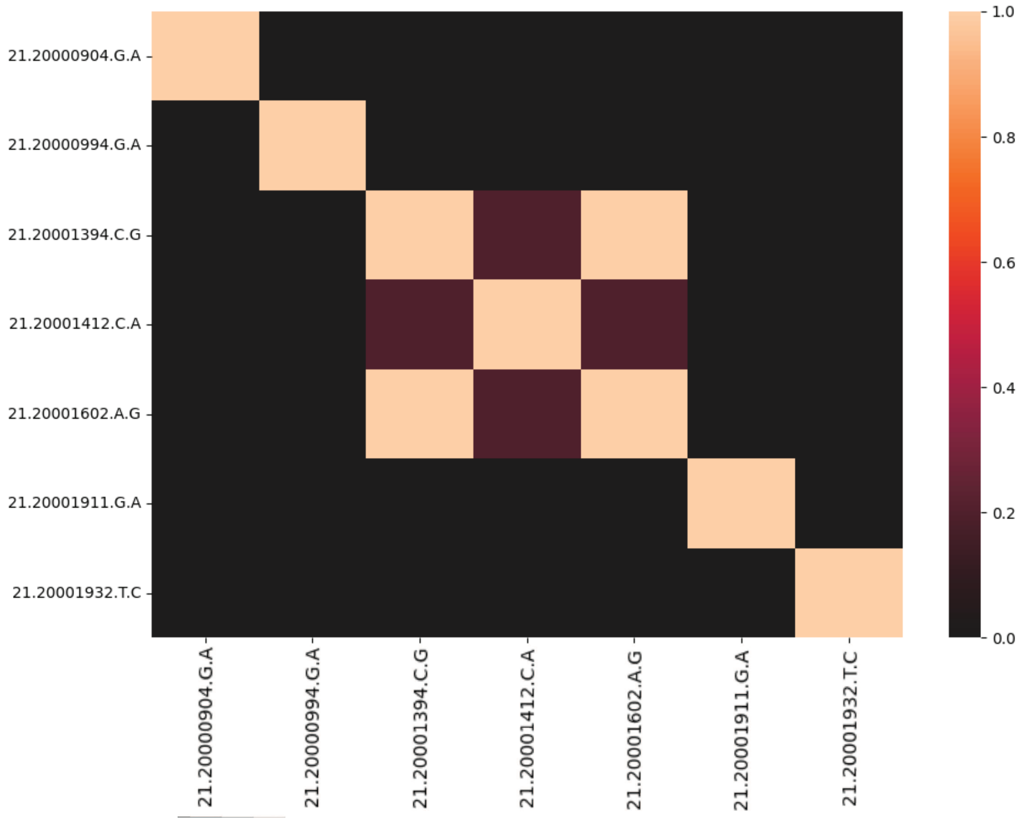

Fig. 3. Example LD submatrix heapmat generated from chr21 LD data.

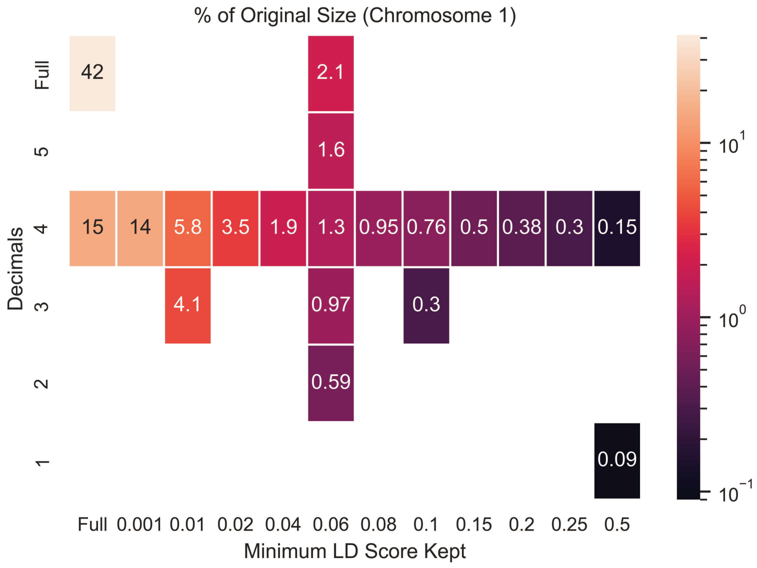

Fig. 4. The amount of compression gained at different parameters for Chromosome 1.

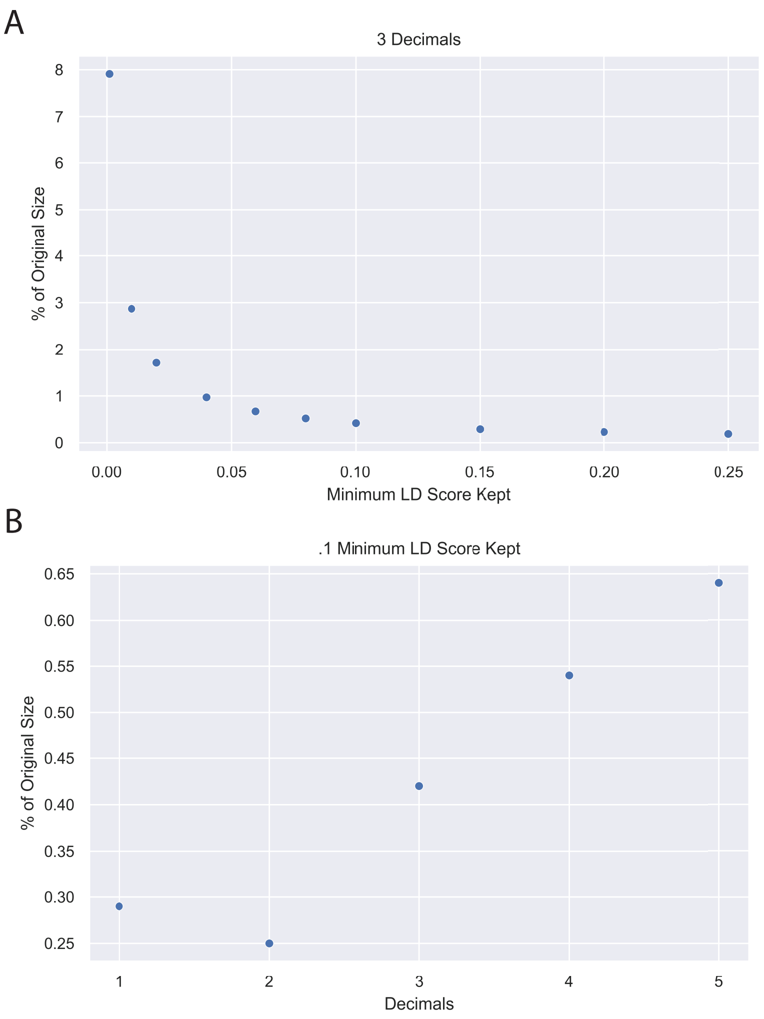

Fig. 5. (A) Compression ratio as a function of minimum LD score threshold when 3 decimal points are kept. (B) Compression ratio as a function of number of decimal points to keep when the minimum LD score threshold is 0.1.

```
Usage: ldmat [OPTIONS] COMMAND [ARGS]...

  A set of commands for more efficiently storing and querying linkage
  disequilibrium matrices.

Options:
  -l, --log-level [warning|info|debug]
  -h, --help                        Show this message and exit.

Commands:
  convert             compress a single file
  convert-chromosome  compress a bunch of files
  convert-maf         add MAF values to an existing file
  submatrix           select by range of positions
  submatrix-by-list   select by list of positions
  submatrix-by-maf    select by range of MAF values
```

Fig. 6. The CLI for ldmat.

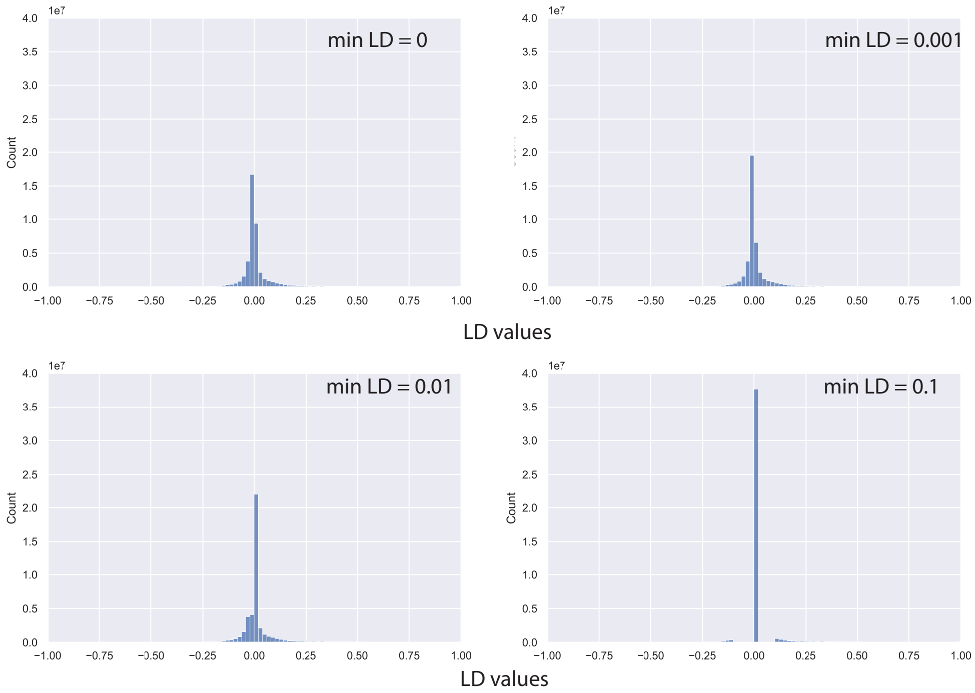

Fig. 7. Distribution of LD values after different minimum LD value threshold.

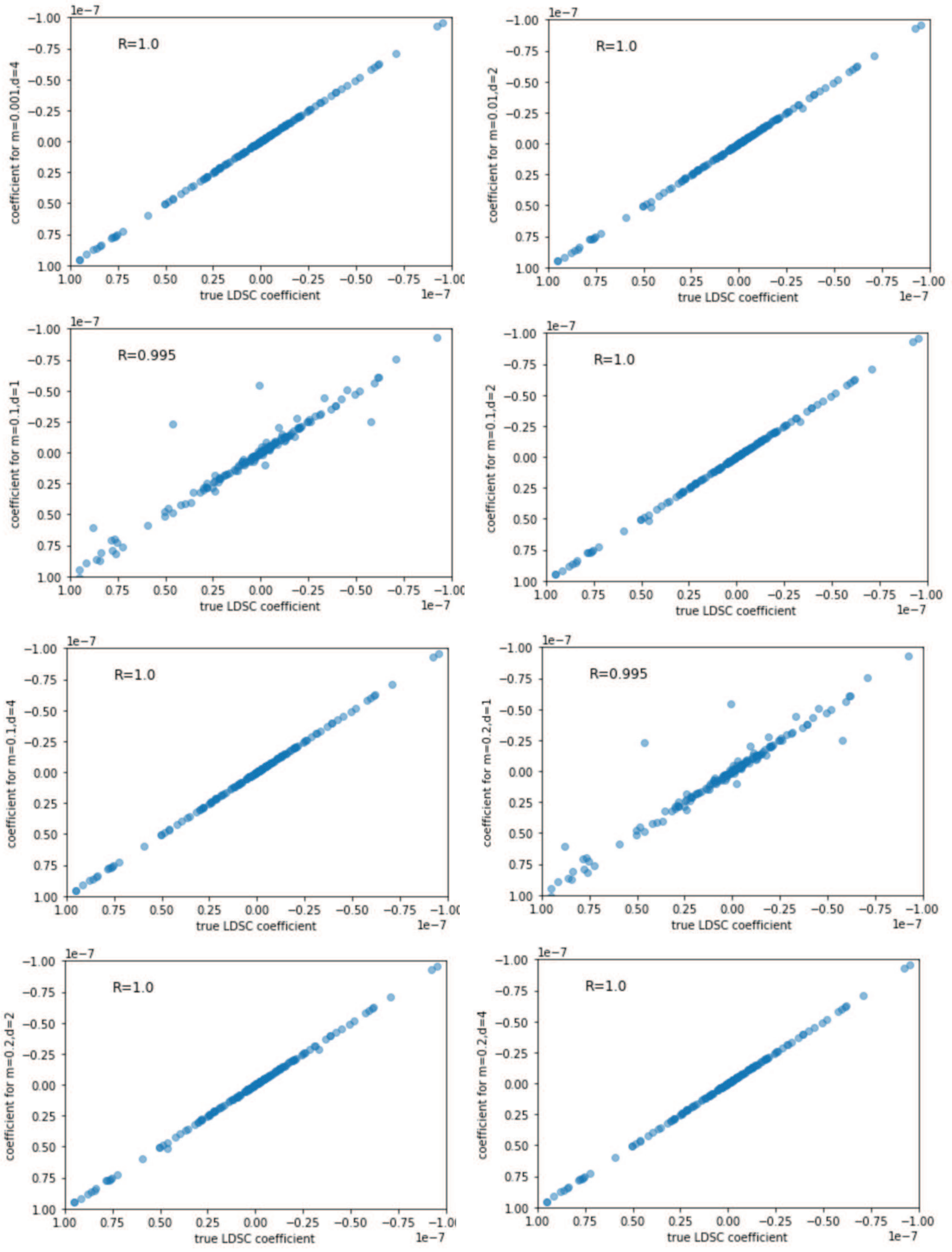

Fig. 8. The correlation between original matrices and matrices derived from different minimum LD thresholds (m) and decimal place thresholds (d) for the LDSC coefficients per annotation. In all minimum LD thresholds tested, four decimal place cut-off gives the most accurate LDSC results.
